# *O*-GlcNAc modification of GSDMD attenuates LPS-induced endothelial cells pyroptosis

**DOI:** 10.1007/s00011-023-01812-1

**Published:** 2023-11-14

**Authors:** Fan Yu, Zhen Zhang, Yiping Leng, Alex F. Chen

**Affiliations:** 1 Department of Cardiology, The Third Xiangya Hospital of Central South University, Changsha, China; 2 Research Center for Life Science and Human Health, Binjiang Institute of Zhejiang University, Hangzhou, Zhejiang China; 3 The Affiliated Changsha Central Hospital, Research Center for Phase I Clinical Trials, Hengyang Medical School, University of South China, Changsha, Hunan China; 4 Department of Cardiology, Institute for Cardiovascular Development and Regenerative Medicine, Xinhua Hospital Affiliated to Shanghai Jiaotong University School of Medicine, 1665 Kongjiang Road, Shanghai, 200092 China

**Keywords:** GSDMD, *O*-GlcNAc, Sepsis, Endothelial, Pyroptosis

## Abstract

**Objective:**

Increased *O*-linked β-*N*-acetylglucosamine (*O*-GlcNAc) stimulation has been reported to protect against sepsis associated mortality and cardiovascular derangement. Previous studies, including our own research, have indicated that gasdermin-D(GSDMD)-mediated endothelial cells pyroptosis contributes to sepsis-associated endothelial injury. This study explored the functions and mechanisms of *O*-GlcNAc modification on lipopolysaccharide (LPS)-induced pyroptosis and its effects on the function of GSDMD.

**Methods:**

A LPS-induced septic mouse model administrated with *O*-GlcNAcase (OGA) inhibitor thiamet-G (TMG) was used to assess the effects of *O*-GlcNAcylation on sepsis-associated vascular dysfunction and pyroptosis. We conducted experiments on human umbilical vein endothelial cells (HUVECs) by challenging them with LPS and TMG to investigate the impact of *O*-GlcNAcylation on endothelial cell pyroptosis and implications of GSDMD. Additionally, we identified potential *O*-GlcNAcylation sites in GSDMD by utilizing four public *O*-GlcNAcylation site prediction database, and these sites were ultimately established through gene mutation.

**Results:**

Septic mice with increased *O*-GlcNAc stimulation exhibited reduced endothelial injury, GSDMD cleavage (a marker of pyroptosis). *O*-GlcNAc modification of GSDMD mitigates LPS-induced pyroptosis in endothelial cells by preventing its interaction with caspase-11 (a human homologous of caspases-4/5). We also identified GSDMD Serine 338 (S338) as a novel site of *O*-GlcNAc modification, leading to decreased association with caspases-4 in HEK293T cells.

**Conclusions:**

Our findings identified a novel post-translational modification of GSDMD and elucidated the *O*-GlcNAcylation of GSDMD inhibits LPS-induced endothelial injury, suggesting that *O*-GlcNAc modification-based treatments could serve as potential interventions for sepsis-associated vascular endothelial injury.

## Introduction

Vascular endothelial injury is a critical factor in the pathogenesis and progression of sepsis [1–4]. This injury is characterized by the disruption of the endothelial barrier, increased vascular permeability, and leakage, which ultimately results in reduced blood flow perfusion in the microcirculation [2]. Existing literature has reported a close association between sepsis and vascular inflammation, thrombosis, and decreased blood perfusion, all of which contribute to increased mortality in septic patients. Evidence from evidence-based medicine shows that anti-inflammatory and anti-thrombotic therapies alone do not improve the ultimate outcomes of septic patients [5, 6]. Therefore, in our study, the focus has been on elucidating the potential mechanisms underlying the decrease in blood perfusion in sepsis. Previous research, including studies conducted by others and our own, has demonstrated that endothelial cell pyroptosis contributes to sepsis-associated endothelial injury and hypoperfusion in sepsis [7–10].

Pyroptosis is an inflammatory type of programmed cell death that triggered intracellular pathogen- or host-derived perturbations of the cytosol, such as lipopolysaccharide (LPS) [11]. Gasdermin-D (GSDMD) acts as an executioner of pyroptosis and is activated by Caspase-11 (a human homologous of Caspases-4/5) during sepsis [12, 13]. Several studies have demonstrated that genetic deficiency of both GSDMD and Caspase-11 protects mice from LPS-induced septic death [9, 14, 15]. The cleavage of GSDMD and subsequent oligomerization of its N-terminal domain on the cell membrane are crucial steps leading to membrane perforation, pyroptosis, and the release of inflammatory factors. Interventions that target the cleavage process of GSDMD may hold therapeutic potential for pyroptosis-related diseases [16, 17]. The ability of activated Caspase-11 to recognize and cleave GSDMD has been extensively reported. Auto-processed Caspase-11 mediates high-affinity binding to the GSDMD-C domain, leading to a tetrapeptide sequence-independent cleavage and the induction of pyroptosis [18]. However, the regulatory mechanism underlying the Caspase-11-GSDMD interaction remains unclear.

In our previous research, we have already demonstrated that LPS can lead to a reduction in blood flow perfusion. The potential mechanism behind this effect may involve an increase in vascular permeability caused by endothelial cell pyroptosis. In this preliminary study, we found that LPS promotes the translocation of GSDMD-N from the cytoplasm to the cell membrane [8]. However, the mechanisms underlying the generation of GSDMD-N have not been thoroughly investigated. Post-translational protein modification is currently at the forefront of research into GSDMD activation [19]. Some studies have shown that succination of GSDMD prevents its interaction with Caspases, thereby limiting its processing, oligomerization, and its ability to induce cell death. *O*-linked β-N-acetylglucosaminylation (*O*-GlcNAcylation) is a highly regulated process that influences protein–protein interactions [20–23]. Recent studies have indicated that increased *O*-GlcNAcylation can mitigate sepsis-associated cardiovascular dysfunction [24–28]. Another study reported that *O*-GlcNAcylation of the kinase RIPK3 blocks protein interactions mediated by the RHIM domain and downstream signaling activation [21]. Therefore, in this study, we aim to investigate the impact of *O*-GlcNAc modification on the interaction between Caspase-4 and GSDMD, as well as its influence on the generation of GSDMD-N.

*O*-GlcNAc modification is tightly regulated by multiple enzymes with opposing effects, namely *O*-GlcNAc transferase (OGT) and *O*-GlcNAcase (OGA), which play a direct role in this modification process [29, 30]. OGT catalyzes the addition of N-acetylglucosamine to serine and threonine residues of target proteins [31], whereas OGA facilitates the hydrolytic removal of *O*-GlcNAc from proteins [32, 33]. Thiamet-G (TMG), an inhibitor of OGA, has been shown to alleviate cardiovascular disorders induced by LPS by increasing *O*-GlcNAc modification [26, 34]. Thus, in our investigation, we will employ OGA inhibitors to explore the role and underlying mechanisms of *O*-GlcNAc modification in sepsis-induced endothelial cell pyroptosis.

In this study, we aimed to elucidate the impact and mechanism of *O*-GlcNAc modification on GSDMD, specifically in inhibiting LPS-induced endothelial cells pyroptosis. These findings have the potential to uncover novel molecular mechanisms and identify promising targets for the treatment of sepsis.

## Materials and methods

### Animals

Male C57BL/6J mice aged 8–10 weeks and weighing 20–25 g were obtained from Hunan Slack Jingda Experimental Animal Breeding Co., Ltd (Changsha, China). The mice were housed in the Animal Experimental Center of Central South University, with a controlled temperature range of 22–24 °C, humidity, and a 12-h light/dark cycle. They were provided with standard diet and water ad libitum. All animal protocols were conducted in accordance with the guidelines for experimental animal welfare ethics established by the Chinese Society of Laboratory Animals. The care of animals and experimental procedures were approved by the Animal Experimental Center of Central South University.

### Experimental animal models

Polymicrobial sepsis was induced by administering a single intraperitoneal dose of lipopolysaccharide (LPS) from Escherichia coli 0111:B4 (Sigma Chemical, MO, United States) at a dosage of 20 mg/kg. To investigate the effects of acute *O*-GlcNAc increase in mice with LPS-induced sepsis, Thiamet-G (TMG) was administered intravenously at a dosage of 600 µg/kg, 12 h prior to LPS treatment. Appropriate control groups were included, with vehicle administration (for LPS and TMG) at the corresponding time points. The animals were randomly assigned to one of the following four groups: saline control (Control), vehicle administration followed by LPS treatment (LPS), TMG administration followed by LPS treatment (LPS + TMG), and TMG administration followed by saline treatment (TMG).

### Measurement of organ blood perfusion

Ten hours after intraperitoneal injection of LPS, we assessed blood flow perfusion in the liver, mesentery, and lower limb using laser Doppler imaging. The procedure for measuring organ blood flow perfusion was conducted as previously described [8]. Mice were anesthetized with intraperitoneal pentobarbital sodium at a dosage of 20 mg/kg body weight. They were then placed on a light-absorbing pad with an electric heating blanket set to maintain a temperature of 37 °C. Laser Doppler imaging was performed using the PeriScan PIM3 system from Somnotec (Singapore), following the manufacturer's instructions.

To measure blood perfusion, a midline abdominal incision was made, and the right lobe of the liver was carefully exposed. Laser Doppler imaging was used to monitor blood flow in this region. Additionally, a selected area of the mesentery was monitored for blood flow. Throughout the procedure, the mice's core temperature was maintained at 37 °C using a heat lamp.

### Cell culture and stimulation

Human umbilical vein endothelial cell lines (HUVECs) and human kidney fibroblast HEK 293T cells were obtained from the American Type Culture Collection (ATCC). HUVECs were cultured in DMEM-F12 medium, while HEK 293T cells were cultured in DMEM. Both culture media were supplemented with 10% fetal bovine serum (FBS) and 1% penicillin–streptomycin. The cells were maintained at 37 °C in a humidified atmosphere containing 5% CO2. HUVECs were plated on six-well plates and exposed to a series of LPS solutions (0, 0.2, 0.4, 0.6, 0.8, 1.0 µg/ml) obtained from InvivoGen (# tlrl-3pelps) for 24 h. The protein levels of O-GlcNAc and GSDMD were assessed by western blotting.

### Lactate dehydrogenase assay

The HUVECs were seeded in a 6-well culture plate one day prior to the experiment and incubated for 24 h. Subsequently, the cells were transferred to 96-well plates. For transfection, the cells were treated with Lipofectamine 2000 reagent (Invitrogen) and transfected with 1 µg/ml of LPS. The transfection was allowed to proceed for 24 h. Cell death was measured using a lactate dehydrogenase (LDH) assay obtained from Abcam, following the instructions provided by the manufacturer.

### Immunofluorescence staining

To prepare frozen aorta tissues, an optimum cutting temperature compound was injected gently through the trachea to maintain fluid in the lungs. The aorta tissues were then frozen, and sections of 5 µm thickness were cut using a cryostat microtome. These sections were mounted on microscope slides. The slides were fixed with 4% paraformaldehyde to preserve the tissue structure and permeabilized with 0.1% Triton X-100 to allow antibody penetration. Primary antibodies specific to CD31 were applied to the slides, followed by incubation with fluorescence-conjugated secondary antibodies. The slides were visualized under a Zeiss fluorescence microscope at a magnification of 40×, and images were captured for further analysis and evaluation.

### Co-immunoprecipitation assay

To lyse HUVECs, cell lysis buffer for Western blot and IP (P0013, Beyotime Biotechnology, Shanghai, China) was used. The cell extracts were treated with Protein A/G agarose beads (Santa Cruz Biotechnology, sc-2003) to remove nonspecifically bound proteins during the immunoprecipitation process. The beads were pelleted by centrifugation, and the supernatant (cell lysate) was transferred to a fresh centrifuge tube kept on ice. Protein A/G agarose beads were added to the cell lysate again, and immunoprecipitation was performed using either anti-GSDMD or anti-caspase-4 antibodies overnight at 4 ºC. The immunoprecipitates were collected by centrifugation, and the supernatant was carefully aspirated and discarded. The pellet was washed with 200 µl of wash buffer. After the final wash, the supernatant was aspirated and discarded, and the pellet was resuspended in 40–60 µl of 1 × electrophoresis sample buffer. The resuspended pellet was then subjected to Western blot analysis using primary antibodies specific to *O*-GlcNAc or GSDMD, respectively.

### GSDMD *O*-GlcNAcylation site prediction

To predict the *O*-GlcNAcylation site of GSDMD, four *O*-GlcNAcylation site prediction sites were utilized. These prediction sites include the DictyOGlyc 1.1 server (http://www.cbs.dtu.dk/services/DictyOGlyc/) [35], NetOGlyc 4.0 server (http://www.cbs.dtu.dk/services/NetOGlyc/) [36], GPP prediction server (https://comp.chem.nottingham.ac.uk/cgi-bin/glyco/bin/getparams.cgi) [37], and GlycoMine (https://glycomine.erc.monash.edu/Lab/GlycoMine/#webserver) [38]. The raw data obtained from these four prediction sites underwent statistical analysis. A Venn diagram was created to determine the predicted set of sites that were identified as the most likely potential *O*-GlcNAcylation sites for GSDMD. This analysis allowed for a comprehensive evaluation of the predictions made by each site and the identification of common sites predicted by multiple prediction methods.

### Plasmid and molecular cloning

The pCMV vector expressing Myc-tagged human OGT (PPL00887-2a) was purchased from PPL. To generate the GSDMD constructs, the gene sequence of GSDMD was obtained from the GenBank database. The template for cloning and construction experiments was obtained, and the necessary modifications were made. The FLAG-tagged GSDMD, FLAG-tagged human GSDMD-NT, FLAG-tagged human GSDMD (S338A) and human GSDMD (S480A) constructs were generated by GENECHEM. The cloning and construction experiments allowed for the creation of the desired mutant plasmids.

### Cell transfection

HEK293T and HUVECs were cultured in DMEM or F-12 medium supplemented with 10% FBS, 100 U/ml penicillin, and 100 µg/ml streptomycin, respectively. The cells were incubated in a humidified atmosphere containing 5% CO_2_. For transfection, cells were grown in 6-well plates until they reached 90% confluence. Plasmid DNA was added to 500 µl of medium, while Lipofectamine 2000 transfection reagent (Invitrogen) was added to another 500 µl of medium. After incubating for 5 min at room temperature, the two media were mixed and incubated for an additional 20 min. The cell culture medium was then replaced with the mixture of media containing the plasmid and Lipofectamine 2000.

### Immunoblotting

Cultured HUVECs were collected and lysed with lysis buffer containing 98% RIPA lysis buffer, 1% protease inhibitor, and 1% phosphatase inhibitor. The cells were incubated with the lysis buffer on ice for 30 min. The total protein concentration in the lysate was measured using the BCA protein assay kit from Beyotime Biotechnology, Shanghai, China. Equal amounts of protein from each sample were loaded onto SDS-PAGE gels and separated by electrophoresis. The proteins were then transferred onto polyvinylidene fluoride (PVDF) membranes and blocked with PBST containing 5% skim milk blocking buffer at room temperature for 2 h.

Primary antibodies, including *O*-GlcNAc (Cell Signaling Technology, #9875), GSDMD (Santa Cruz Biotechnology, sc-393581), GSDMD (Abcam, ab209845), β-actin (SAB, #21338), GAPDH (Proteintech), OGT (Santa Cruz Biotechnology, sc-74546), Myc Tag (Invitrogen, # R951-25), OGA (Proteintech, 14711-1-AP), were incubated with the membranes followed by incubation with HRP-conjugated secondary antibodies (anti-rabbit IgG from Bioword, and anti-mouse IgG from Bioword). Protein bands were detected using an Enhanced Chemiluminescent (ECL) reagent from Thermo Scientific, and the images were acquired with a ChemiDoc MP System from Bio-Rad. Densitometric analysis was performed using ImageJ Software to quantify the protein bands.

### Statistics

All experiments were conducted with a minimum of three independent replicates to ensure robustness and reliability of the results. Statistical analysis was performed using GraphPad Prism 8 software. The data are presented as mean ± standard deviation (SD). To compare the mean values of biochemical data between different groups, a two-tailed Student’s *t* test was employed. For comparisons involving multiple time points, repeated-measures analysis of variance (ANOVA) was used, followed by Bonferroni post-tests. A significance level of *P* < 0.05 was considered statistically significant for all analyses.

## Results

### *O*-GlcNAc modification ameliorated vascular endothelial injury in septic mice

**Fig. 1:**
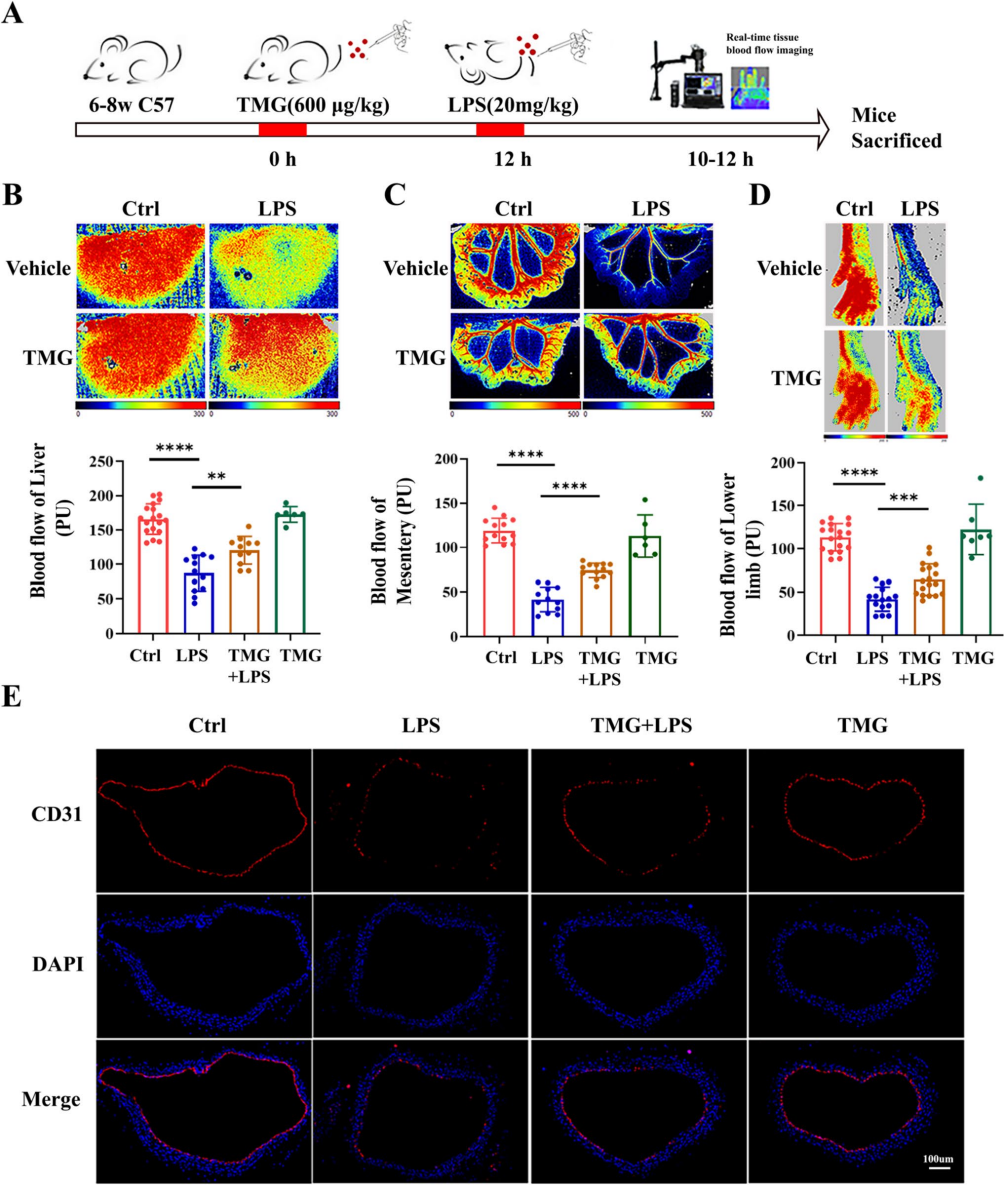
O-GlcNAc modification ameliorated vascular endothelial injury in septic mice. **A** Experimental design for assessing the effect of Thiamet-G(TMG) on LPS-induced blood flow perfusion. 600 μg/kg TMG were injected intravenously 12 h before injection of LPS (20 mg/kg) was applied 10–12 h. **B**–**D** Analysis of blood flow perfusion of the liver, mesentery, and lower limbs by laser Doppler imaging in different treatment mice at 12 h after induction of endotoxemia (20 mg/kg i.p.). **E** Immunofluorescence analysis of endothelial marker CD31 in the aorta of different treatment mice at 12 h after induction of endotoxemia (20 mg/kg i.p.). Scale bar: 100 μm, *n* = 5, Data are expressed as means ± SD of six animals per treatment, **p < 0.005, ***p < 0.0005, ****p < 0.0001

Following the methods described in the literature [26], we administered the OGA inhibitor TMG for 12 h, followed by intraperitoneal injection of LPS for an additional 12 h. We then assessed the blood flow perfusion in the liver, mesentery, and lower limbs of the mice using Laser Doppler imaging (Fig. 1A). After LPS administration, a significant decrease in blood flow perfusion was observed in the liver, mesentery, and lower limbs (*P* < 0.0001). In contrast, the septic mice treated with TMG showed a significant increase in blood perfusion in these regions compared to the sepsis group (LPS) (*P* < 0.005) (Fig. 1B–D). To investigate the impact of *O*-GlcNAc modification on the inhibition of vascular endothelial injury, we examined the expression of the vascular endothelial marker CD31 in the mice aorta. The results revealed a reduction in the fluorescence expression of CD31 in the vascular endothelial of septic mice compared to control mice. However, treatment with TMG effectively mitigated the LPS-induced reduction in the endothelial marker CD31 (Fig. 1E). These findings suggest that TMG has the potential to alleviate vascular endothelial injury induced by LPS.

### Increased *O*-GlcNAc levels inhibited GSDMD cleavage in the aorta of septic mice

**Fig. 2:**
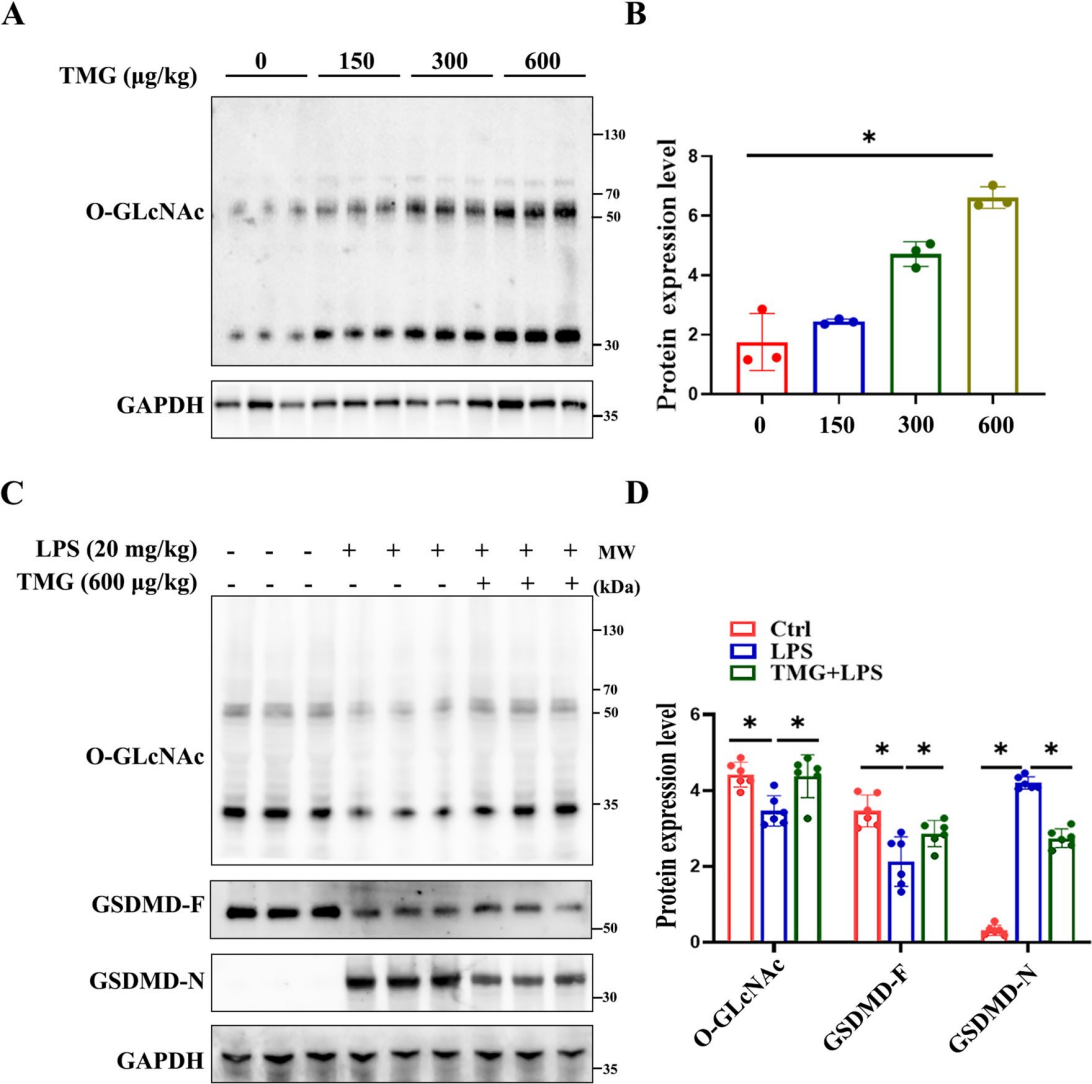
Increased *O*-GlcNAc levels inhibited GSDMD cleavage. **A**, **B** Western Blot analysis of different doses of TMG were injected intravenously 12 h to detect the global *O*-GLcNAc level. Data are expressed as means ± SD of three animals per treatment, *p < 0.05, *n* = 3. **C**, **D** Western Blot analysis of TMG (600 μg/kg) were injected intravenously 12 h following injection of LPS (20 mg/kg) for 12 h to detect the global O-GlcNAc level and GSDMD-N production. Data are expressed as means ± SD of six animals per treatment, *p < 0.05, *n* = 6

To elucidate the dose-dependent effect of TMG on increasing total vascular protein *O*-GlcNAcylation levels, we initially administered different doses of TMG via tail vein injection. Subsequently, 12 h post-injection, Western blot analysis was conducted to assess vascular total protein *O*-GlcNAc expression. The results indicated that a dose of 600 µg/kg TMG exhibited a consistently stable increase in vascular *O*-GlcNAc levels (Fig. 2A, B). Subsequent experiments were performed using this dosage condition. To assess the impact of TMG treatment on GSDMD protein cleavage, we examined the levels of global *O*-GlcNAc and GSDMD in the mice aorta. In an experimental sepsis model induced by LPS, aorta samples exhibited a decrease in global *O*-GlcNAc levels, accompanied by a significant increase in GSDMD-N production. However, upon treatment with TMG, the aorta showed increased global *O*-GlcNAc levels and decreased levels of GSDMD-N, as demonstrated by Western blot analysis (Fig. 2C, D). These findings demonstrate that increased *O*-GlcNAc levels contribute to the reduction of endothelial cell pyroptosis in septic mice.

### Increased *O*-GlcNAc modification inhibited LPS-induced endothelial cells pyroptosis

**Fig. 3:**
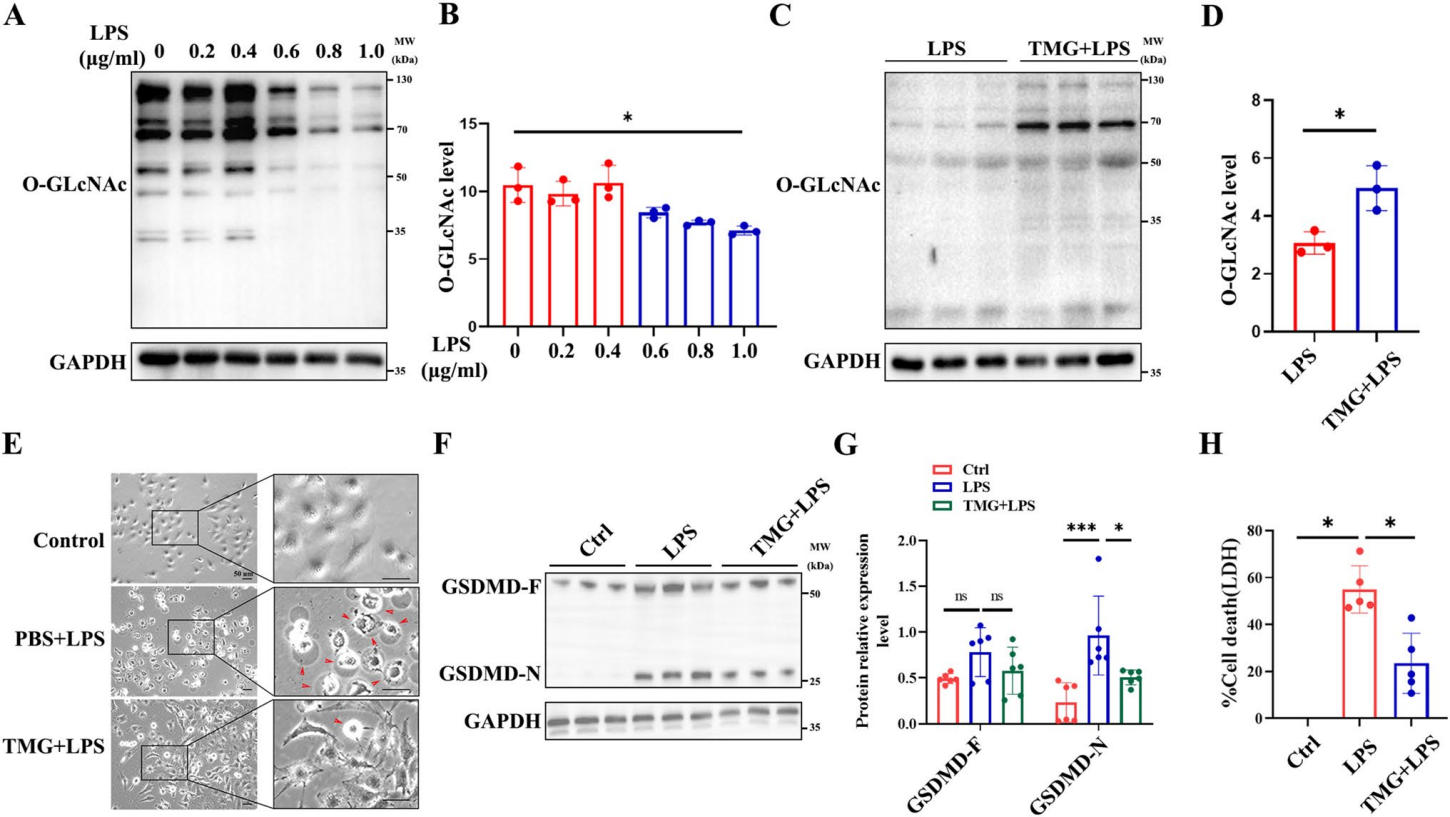
*O*-GlcNAc modification inhibits LPS-induced endothelial cells pyroptosis. **A**, **B** HUVECs transfected with LPS induced marked *O*-GLcNAc protein level in a concentration-dependent manner and statistics analysis of *O*-GLcNAc level, *n* = 3. **C**, **D** Western Blott showing global *O*-GlcNAc levels under TMG and LPS treatment, statistics analysis of O-GLcNAc level, *n* = 3. **E** Total cell lysates of HUVECs untreated or stimulated with LPS (1 μg/ml) alone with or without TMG (1 μM) pretreatment marked cell swell and lysis, *n* = 3. Scale bar: 50 μm, *n* = 3. **F**, **G** TMG (1 μM) treated HUVECs transfected with LPS induced marked GSDMD cleavage decreased as compared with that in HUVECs transfected with LPS, Statistics analysis of GSDMD-F and GSDMD-N level, *n* = 6. **H** TMG (1 μM) treated HUVECs transfected with LPS induced marked LDH release decreased as compared with that in HUVECs transfected with LPS, *n* = 3. Data are expressed as means ± SD of three independent experiments, *p < 0.05

To further investigate the role of *O*-GlcNAc modification in regulating LPS-induced endothelial cell pyroptosis in vitro, we treated HUVECs with different concentrations of LPS and examined the global *O*-GlcNAc levels. As shown in Fig. 3A, B, the global *O*-GlcNAc levels decreased with increasing LPS stimulation concentration. However, administration of TMG increased the global *O*-GlcNAc levels in HUVECs (Fig. 3C, D). Furthermore, we evaluated the effects of *O*-GlcNAc modification on LPS-induced pyroptosis. HUVECs treated with LPS exhibited rapid morphological changes and LDH release, indicating the occurrence of pyroptosis. However, these effects were inhibited by TMG treatment (Fig. 3E, H). Additionally, TMG pretreatment reduced the levels of GSDMD-N protein, as shown in Fig. 3F, G, suggesting that TMG may exert its effects by inhibiting GSDMD cleavage. In summary, these findings collectively demonstrate that *O*-GlcNAc modification inhibits LPS-induced endothelial cell pyroptosis, providing further evidence for the role of *O*-GlcNAc in regulating inflammatory processes in endothelial cells.

### GSDMD *O*-GlcNAcylation involvement in LPS-induced endothelial cell pyroptosis

**Fig. 4:**
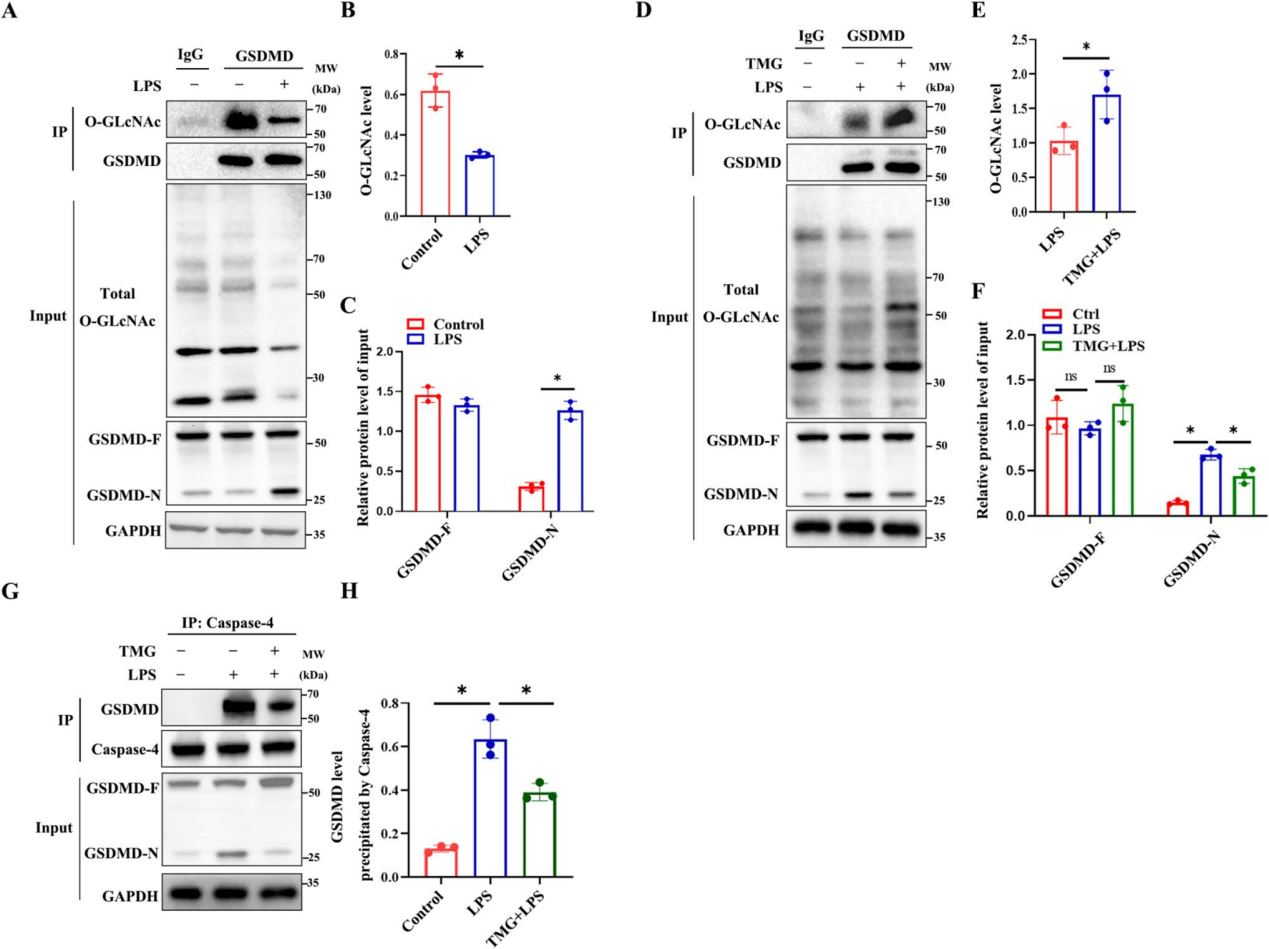
GSDMD *O*-GlcNAcylation involvement in LPS-induced endothelial cell pyroptosis. **A** Total cell lysates of HUVECs untreated or stimulated with LPS. Immunoprecipitation with anti-GSDMD antibody to pull down *O*-GlcNAcylated proteins, *n* = 3. **B** Total cell lysates of HUVECs untreated or stimulated with LPS alone with or without TMG pretreatment. Immunoprecipitation with anti-GSDMD antibody to pull down *O*-GlcNAcylated proteins, *n* = 3. **C** Total cell lysates of HUVECs untreated or stimulated with LPS alone with or without TMG pretreatment was immunoprecipitated with anti-Caspase-4 antibody, followed by immunoblotting with anti-GSDMD antibody, *n* = 3. **D** Statistics analysis of GSDMD level precipitated by Caspase-4. **E** Statistics analysis of *O*-GlcNAc level precipitated by GSDMD under untreated or stimulated with LPS. **F** Statistics analysis of GSDMD-F and GSDMD-N level of input group under untreated or stimulated with LPS. **G** Statistics analysis of *O*-GlcNAc level precipitated by GSDMD under untreated or stimulated with LPS alone with or without TMG pretreatment. **H** Statistics analysis of GSDMD-F and GSDMD-N level of input group under untreated or stimulated with LPS alone with or without TMG pretreatment. Data are expressed as means ± SD of three independent experiments, *p < 0.05

To investigate the *O*-GlcNAcylation level of GSDMD and the effect of *O*-GlcNAc modification on the interaction between Caspase-4 and GSDMD under LPS stimulation in HUVECs, we conducted a co-immunoprecipitation (Co-IP) experiment. Firstly, we performed a Co-IP experiment between *O*-GlcNAc and GSDMD to observe the impact of LPS on GSDMD *O*-GlcNAcylation. (Fig. 4A–C). And then, when TMG was administered under LPS treatment, a Co-IP experiment between *O*-GlcNAc and GSDMD to observe the impact of LPS and TMG on GSDMD *O*-GlcNAcylation, it increased the *O*-GlcNAcylation of GSDMD and decreased the expression of GSDMD-N (Fig. 4D–F). The results suggest that *O*-GlcNAcylation of GSDMD may be involved in the regulation of pyroptosis effector molecule GSDMD-N expression by LPS and TMG. Furthermore, we used Caspase-4 antibodies for Co-IP and found that the interaction between Caspase-4 and GSDMD increased under LPS stimulation. However, this interaction was inhibited by TMG administration (Fig. 4G–H). These findings suggest that *O*-GlcNAcylation of GSDMD involvement in LPS-induced endothelial cell pyroptosis.

### Increased *O*-GlcNAc modification of GSDMD Ser-338 lead to a reduction in interaction of GSDMD and Caspase-4

**Fig. 5:**
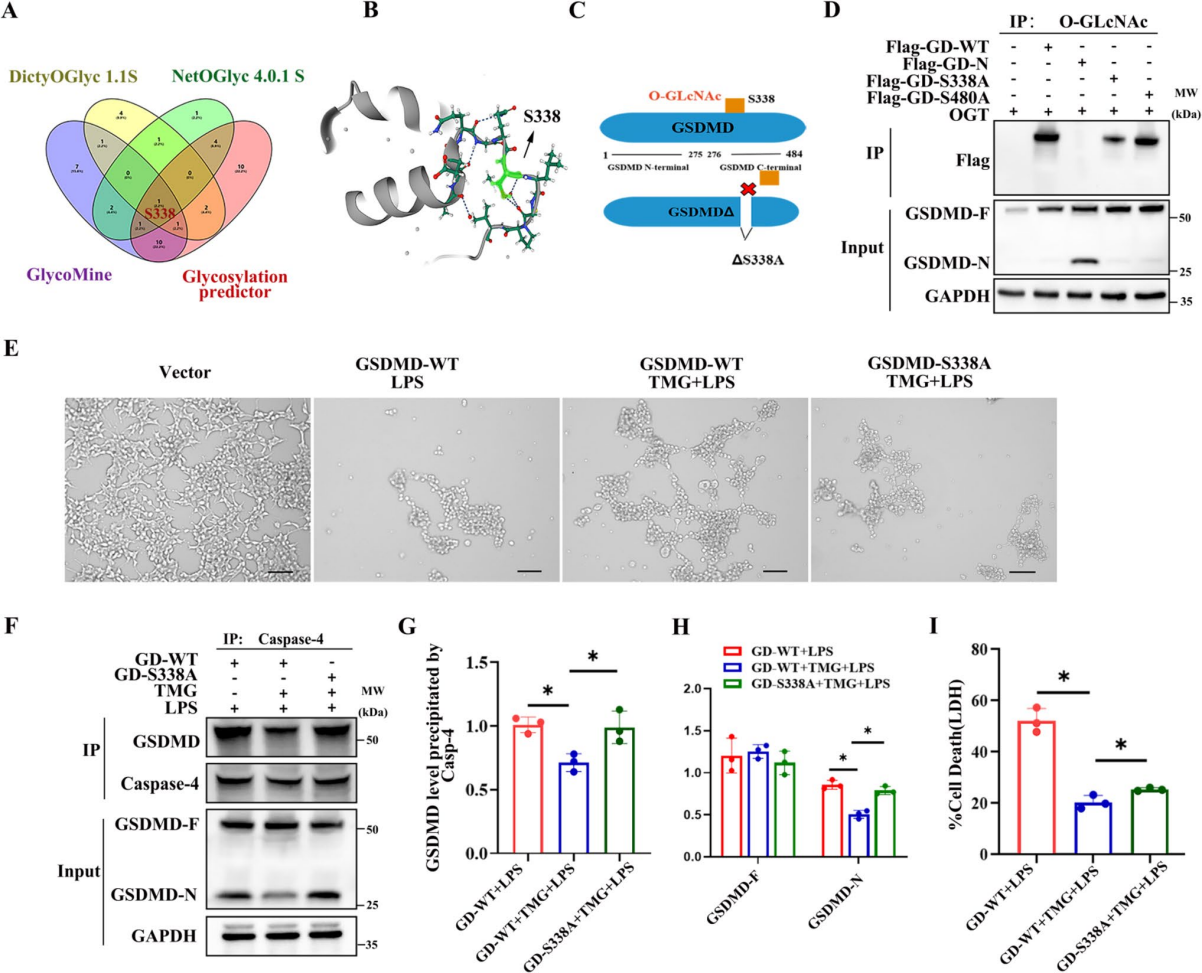
Increased *O*-GlcNAc modification of GSDMD Ser338 lead to a reduction in interaction of GSDMD and caspase-4. **A** Venn analysis diagram of *O*-GlcNAcylation prediction websites. **B**, **C** A view of the GSDMD mutant site. **D** *O*-GlcNAcylation of GSDMD-WT, GSDMD-S338A and GSDMD-S480A overexpressed in 293T cells in the presence of OGT, *n* = 3. **E** Cell morphology change of HUVECs untreated or stimulated with LPS alone with or without TMG pretreatment. Scale bar: 50 μm, *n* = 3. **F** GSDMD and GSDMD S338A mutant overexpressed in 293T cells untreated or stimulated with LPS alone with or without TMG was immunoprecipitated with anti-Caspase-4 antibody, followed by immunoblotting with anti-GSDMD antibody, *n* = 3. **G** Statistics Analysis of GSDMD level of immunoprecipitated with Caspase-4. **H** Statistics Analysis of GSDMD-F and GSDMD-N level of Input group. **I** LDH release of HUVECs untreated or stimulated with LPS alone with or without TMG pretreatment, *n* = 3. Data are expressed as means ± SD of three independent experiments, *p < 0.05

To identify potential *O*-GlcNAc modification sites on GSDMD, multiple prediction websites including DictyOGlyc 1.1 server, NetOGlyc 4.0 server, GPP prediction server, and GlycoMine were employed. Bioinformatic analysis suggests that Ser-338 may be a specific site for GSDMD *O*-GlcNAc modification (Fig. 5A). To confirm the importance of this site, site-directed mutagenesis was performed to generate a single mutant (S338A) within the C-terminal domain of GSDMD (Fig. 5B, C). In HEK293T cells, Flag-GSDMD-WT, Flag-GSDMD-S338A, and Flag-GSDMD-S480A (another potential *O*-GlcNAc modification site) were co-expressed with OGT. Western blot analysis revealed that GSDMD-WT and GSDMD-S480A were modified by *O*-GlcNAc, while GSDMD-S338A showed significantly reduced *O*-GlcNAc modification (Fig. 5D). These results indicated that Ser-338 might be the key site for *O*-GlcNAc modification of GSDMD. Furthermore, it was observed that TMG treatment reversed LPS-induced cell death in HEK293T cells transfected with GSDMD-WT plasmid. However, when GSDMD-S338A was transfected, TMG treatment had no effect on LPS-induced cell death (Fig. 5E). To further investigate the effect of GSDMD *O*-GlcNAcylation on GSDMD-N generation and the interaction between Caspase-4 and GSDMD. We employed the S338A mutant plasmid to perform a Co-IP experiment between Caspase-4 and GSDMD, the results indicate that when GSDMD is able to be *O*-GlcNAc-modified normally (GD-WT), TMG can increase GSDMD *O*-GlcNAcylation, thereby reducing the binding between caspase-4 and GSDMD as well as GSDMD cleavage (Lane 2 vs. Lane 1); GSDMD S338A cannot be *O*-GlcNAcylated (GD-S338A), and the effect of TMG in increasing GSDMD *O*-GlcNAcylation is abolished, resulting in the binding between Caspase-4 and GSDMD as well as GSDMD cleavage (Lane 3 vs. Lane 2) (Fig. 5F–H). Moreover, TMG reduced LDH release under LPS stimulation, while the GSDMD-S338A mutant exhibited defects in LDH release (Fig. 5I). These results suggest that reduced *O*-GlcNAcylation of GSDMD under LPS stimulation conditions promotes pyroptosis.

## Discussion

**Fig. 6:**
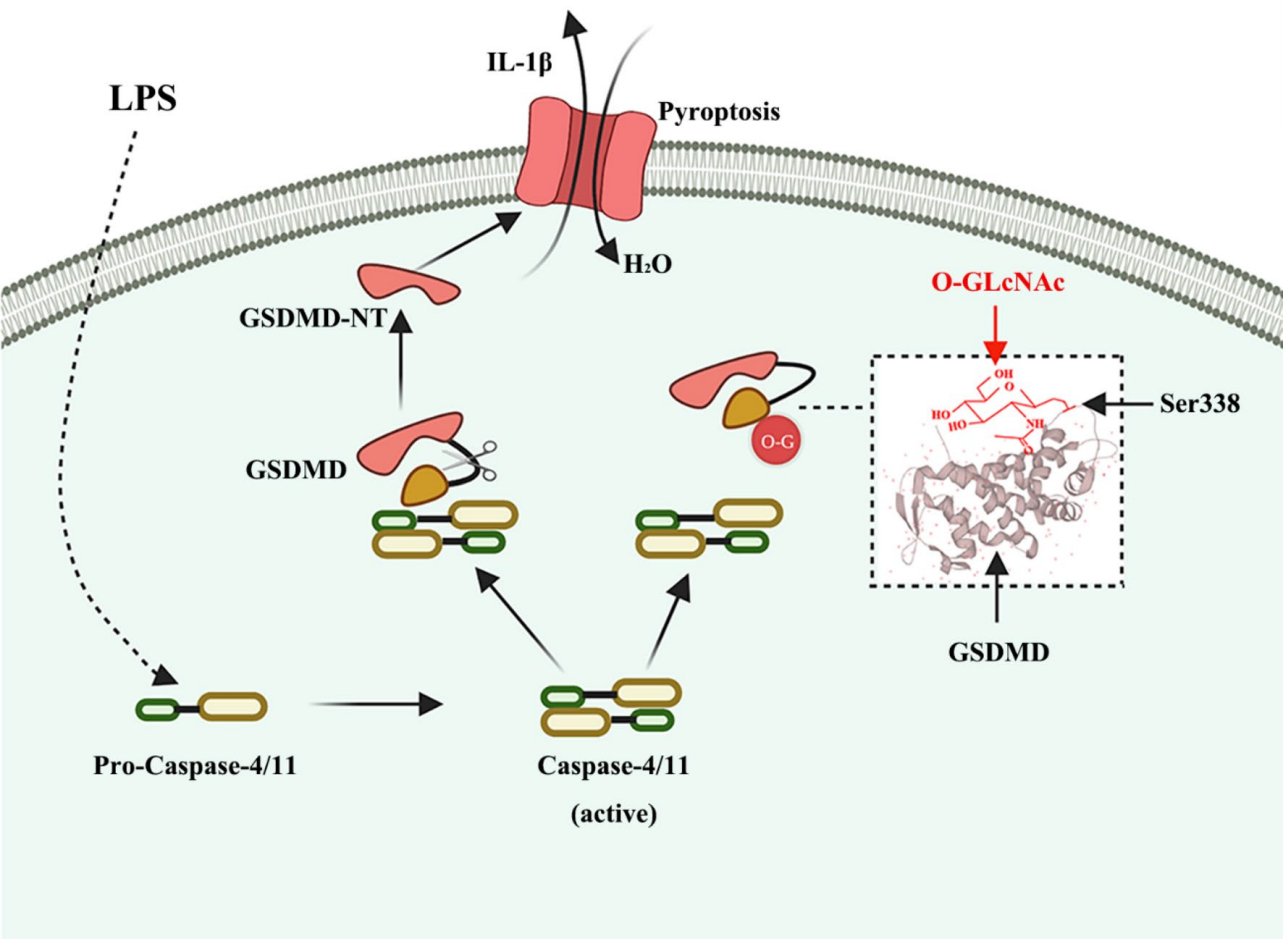
Schema of hypothesis that *O*-GlcNAc modification of GSDMD attenuates LPS-induced endothelial cells pyroptosis. Intracellular LPS directly activates caspase-4/11, and activated caspase-4/11 can cleave GSDMD and induce pyroptosis, which promotes the release of inflammatory factors and the entry of extracellular water into the cell, leading to cell swelling and lysis. While GSDMD can be modified by *O*-GlcNAc under the action of TMG, so that caspase-4/11 cannot bind to GSDMD, thus to prevent the cleavage of GSDMD, and finally inhibit the occurrence of pyroptosis. *O–G* O-GlcNAc

In this study, we uncovered a novel post-translational modification of GSDMD and its functional implications. We provided evidence for the beneficial effects of enhancing *O*-GlcNAc modification on organ blood flow perfusion in sepsis. Our findings indicated that *O*-GlcNAcylation of GSDMD played a crucial role in the regulation of vascular endothelial function. Specifically, *O*-GlcNAcylation of GSDMD suppressed its interaction with caspase-4, leading to a decrease in LPS-induced GSDMD-N formation in HUVECs. The authors further identified Ser-338 as an important *O*-GlcNAcylation site on GSDMD during the process of pyroptosis (Fig. 6). These findings provide new evidence and insights into the mechanisms by which *O*-GlcNAc inhibits pyroptosis.

Our previous study discovered that GSDMD significantly exacerbated multiple-organ perfusion disturbances in endotoxemia models, which paralleled endotoxin death in mice [8]. Recent studies have also reported that acute increase in *O*-GlcNAc levels improves survival in mice with LPS-induced sepsis. In this study, they investigated the protective effect of *O*-GLcNAc on vascular injury in septic mice by constructing a sepsis model using intraperitoneal injection of 20 mg/kg LPS [26]. Thus, our study utilized this modeling approach to investigate the relationship between *O*-GlcNAc levels and vascular injury. We demonstrated that elevated *O*-GlcNAc levels promoted the blood flow perfusion in the liver, mesentery, and lower limbs of the LPS induced septic mice, increased the expression of the vascular endothelial marker CD31 in the LPS induced septic mice aorta, indicating that *O*-GlcNAcylation might improve the prognosis of sepsis by influencing blood flow perfusion and vascular endothelial integrity. Additionally, our findings indicated that TMG treatment inhibited the cleavage of vascular GSDMD in the septic mouse model, suggesting that *O*-GlcNAcylation regulates the process of pyroptosis.

This study, along with others, has demonstrated that increasing *O*-GLcNAc levels can mitigate endothelial injury induced by LPS, TNF-α and other factors [27]. Building upon this knowledge, we conducted further investigations and discovered that *O*-GlcNAcylation of GSDMD plays a crucial role in LPS-induced pyroptosis. The study supports the notion that *O*-GlcNAc modification of GSDMD inhibits LPS-induced endothelial cell pyroptosis by interfering with its interaction with Caspases, thereby preventing its cleavage and subsequent cell death. Other studies have also reported the inhibitory effects of GSDMD succination, another post-translational modification, on LPS-induced pyroptosis [19]. Based on these experimental findings, it is suggested that post-translational modifications of proteins play a protective role in vascular endothelial injury caused by LPS or other injurious factors.

Glycosylation, including *O*-GlcNAcylation, is known to regulate protein–protein interactions [23, 30, 39]. Previous studies have demonstrated that *O*-GlcNAcylation of RIPK3 blocks the interaction between RIPK3 and RIPK1 or RIPK3 and itself, thereby inhibiting downstream signaling activation [21]. Similarly, increased *O*-GlcNAcylation of A20 enhances its binding to TAX1BP1, a regulatory protein for A20 activity [27]. In our study, we found that *O*-GlcNAcylation of GSDMD on Ser-338 inhibits its interaction with Caspase-4. *O*-GlcNAc modification of proteins may induce conformational changes that affect the exposure of specific protein sequences, potentially altering the intermediate junction region of GSDMD or the binding site for Caspases in its C-terminal region. The specific mechanism by which Ser-338 sites of GSDMD affect Caspases-GSDMD interaction will be further investigated in future studies. It's important to note that besides glycosylation, other post-translational modifications such as phosphorylation, acetylation, and ubiquitination can also regulate protein function [40, 41]. Whether these modifications occur in GSDMD and contribute to its functional changes will require further experimental validation.

Although we have identified the role and mechanism of *O*-GlcNAc modification of GSDMD in LPS-induced pyroptosis, a comprehensive review is still needed to fully understand the effects of *O*-GlcNAcylation on pyroptosis. It is important to acknowledge that our study does not exclude the possibility of *O*-GlcNAc modification on GSDME, another protein involved in pyroptosis, and its potential role in regulating cell pyroptosis. GSDMD has been recognized as the primary substrate for Caspases in pyroptosis, particularly in sepsis, while GSDME mediates pyroptosis in other diseases [11]. In a study utilizing mice lacking only Caspase-1, where a Caspase-11 bacterial artificial chromosome transgene was microinjected into Caspase-1/11 double-knockout embryos, the authors demonstrated that Caspase-1-independent noncanonical pathways mediated by Caspase-11 promoted lethal septic shock following LPS challenge. They found that cytosolic LPS-mediated pyroptosis was the main driver for septic shock, highlighting the crucial role of Caspase-4/5/11 as LPS receptors [12].

In conclusion, our study revealed that GSDMD undergoes *O*-GlcNAc modification, and this modification plays a crucial role in suppressing pyroptosis by preventing the interaction between Caspases and GSDMD. Moreover, increasing *O*-GlcNAcylation promoted organ blood flow perfusion and protected against vascular endothelial dysfunction in a mouse sepsis model. These findings provide novel insights into the regulatory role of *O*-GlcNAcylation in pyroptosis during bacterial infections. Targeting GSDMD *O*-GlcNAcylation could be a potential therapeutic approach for the treatment of sepsis.

## Data Availability

The datasets used during the present study are available upon reasonable request.

## Abbreviations

*O*-GlcNAc: *O*-Linked β-*N*-acetylglucosamine
GSDMD: Gasdermin-D
LPS: Lipopolysaccharide
HUVECs: Human umbilical vein endothelial cells
OGT: *O*-GlcNAc transferase
OGA: *O*-GlcNAcase
TMG: Thiamet-G
LDH: Lactate dehydrogenase

## References

[1] Darwish I, Liles WC (2013). Emerging therapeutic strategies to prevent infection-related microvascular endothelial activation and dysfunction. Virulence.

[2] Joffre J, Hellman J, Ince C, Ait-Oufella H (2020). Endothelial responses in sepsis. Am J Respir Crit Care Med.

[3] Opal SM, van der Poll T (2015). Endothelial barrier dysfunction in septic shock. J Intern Med.

[4] Lee WL, Slutsky AS (2010). Sepsis and endothelial permeability. N Engl J Med.

[5] Dinarello CA (2000). Proinflammatory cytokines. Chest.

[6] Warren BL, Eid A, Singer P, Pillay SS, Carl P, Novak I, Chalupa P, Atherstone A, Penzes I, Kubler A, Knaub S, Keinecke HO, Schindel F, Heinrichs H, Juers M, Bone RC, Opal SM, KyberSept Trial Study Group (2001). Caring for the critically ill patient. High-dose antithrombin III in severe sepsis: a randomized controlled trial. JAMA.

[7] Chen Q, Yang Y, Hou J, Shu Q, Yin Y, Fu W (2019). Increased gene copy number of DEFA1/DEFA3 worsens sepsis by inducing endothelial pyroptosis. Proc Natl Acad Sci USA.

[8] Liu H, Tang D, Zhou X, Yang X, Lu B, Chen AF (2020). PhospholipaseCγ1/calcium-dependent membranous localization of Gsdmd-N drives endothelial pyroptosis, contributing to lipopolysaccharide-induced fatal outcome. Am J Physiol Heart Circ Physiol.

[9] Cheng KT, Rehman J, Malik AB (2017). Caspase-11–mediated endothelial pyroptosis underlies endotoxemia-induced lung injury. J Clin Invest.

[10] Peng F, Chang W, Sun Q, Xu X, Xie J, Qiu H (2020). HGF alleviates septic endothelial injury by inhibiting pyroptosis via the mTOR signalling pathway. Respir Res.

[11] Shi J, Gao W, Shao F (2017). Pyroptosis: gasdermin-mediated programmed necrotic cell death. Trends Biochem Sci.

[12] Kayagaki N, Stowe IB, Lee BL, O'Rourke K, Anderson K, Warming S (2015). Caspase-11 cleaves gasdermin D for non-canonical inflammasome signalling. Nature.

[13] Yang J, Zhao Y, Shao F (2015). Non-canonical activation of inflammatory caspases by cytosolic LPS in innate immunity. Curr Opin Immunol.

[14] Hu JJ, Liu X, Xia S, Zhang Z, Zhang Y, Zhao J (2020). FDA-approved disulfiram inhibits pyroptosis by blocking gasdermin D pore formation. Nat Immunol.

[15] Rathkey JK, Zhao J, Liu Z, Chen Y, Yang J, Kondolf HC (2018). Chemical disruption of the pyroptotic pore-forming protein gasdermin D inhibits inflammatory cell death and sepsis. Sci Immunol..

[16] Liu Z, Wang C, Yang J, Chen Y, Zhou B, Abbott DW (2020). Caspase-1 engages full-length gasdermin d through two distinct interfaces that mediate caspase recruitment and substrate cleavage. Immunity.

[17] Aglietti RA, Dueber EC (2017). Recent insights into the molecular mechanisms underlying pyroptosis and gasdermin family functions. Trends Immunol.

[18] Wang K, Sun Q, Zhong X, Zeng M, Zeng H, Shi X (2020). Structural mechanism for GSDMD targeting by autoprocessed caspases in pyroptosis. Cell.

[19] Humphries F, Shmuel-Galia L, Ketelut-Carneiro N, Li S, Wang B, Nemmara VV (2020). Succination inactivates gasdermin D and blocks pyroptosis. Science.

[20] Zachara NE (2012). The roles of *O*-linked β-*N*-acetylglucosamine in cardiovascular physiology and disease. Am J Physiol Heart Circ Physiol.

[21] Li X, Gong W, Wang H, Green DR, Singh PK, Wen H (2019). O-GlcNAc transferase suppresses inflammation and necroptosis by targeting receptor-interacting serine_threonine-protein kinase 3. Immunity.

[22] Park J, Ha HJ, Chung ES, Baek SH, Cho Y, Kim HK, Han J, Sul JH, Lee J, Kim E, Kim J, Yang YR (2021). *O*-GlcNAcylation ameliorates the pathological manifestations of Alzheimer’s disease by inhibiting necroptosis. Sci Adv.

[23] Zhang J, Yu P, Hua F, Hu Y, Xiao F, Liu Q, Huang D, Deng F, Wei G, Deng W, Ma J, Zhu W, Zhang J, Yu S (2020). Sevoflurane postconditioning reduces myocardial ischemia reperfusion injury-induced necroptosis by up-regulation of OGT-mediated *O*-GlcNAcylated RIPK3. Aging.

[24] Hilgers RH, Xing D, Gong K, Chen YF, Chatham JC, Oparil S (2012). Acute *O*-GlcNAcylation prevents inflammation-induced vascular dysfunction. Am J Physiol Heart Circ Physiol.

[25] Jensen RV, Andreadou I, Hausenloy DJ, Bøtker HE (2019). The role of *O*-GlcNAcylation for protection against ischemia-reperfusion injury. Int J Mol Sci.

[26] Silva JF, Olivon VC, Mestriner F, Zanotto CZ, Ferreira RG, Ferreira NS (2019). Acute increase in O-GlcNAc improves survival in mice with LPS-induced systemic inflammatory response syndrome. Front Physiol.

[27] Yao D, Xu L, Xu O, Li R, Chen M, Shen H (2018). *O*-Linked β-N-Acetylglucosamine modification of A20 enhances the inhibition of NF-κB (Nuclear Factor-κB) activation and elicits vascular protection after acute endoluminal arterial injury. Arterioscler Thromb Vasc Biol.

[28] Zhong M, Wu W, Wang Y (2020). Inhibition of sphingosine kinase 1 attenuates sepsis-induced microvascular leakage via inhibiting macrophage NLRP3 inflammasome activation in mice. Anesthesiology.

[29] Chatham JC, Young ME, Zhang J (2020). Role of *O*-linked *N*-acetylglucosamine (O-GlcNAc) modification of proteins in diabetic cardiovascular complications. Curr Opin Pharmacol.

[30] Chatham JC, Zhang J, Wende AR (2020). Role of *O*-linked *N*-acetylglucosamine protein modification in cellular (patho)physiology. Physiol Rev..

[31] Ju KE (2020). *O*-GlcNAc transferase: structural characteristics, catalytic mechanism and small-molecule inhibitors. ChemBioChem.

[32] Elbatrawy AA, Kim EJ, Nam G (2020). O-GlcNAcase: emerging mechanism, substrate recognition and small molecule inhibitors. ChemMedChem.

[33] Zachara NE, Hart GW (2002). The emerging significance of O-GlcNAc in cellular regulation. Chem Rev.

[34] Fulop N, Marchase R, Chatham J (2007). Role of protein *O*-linked *N*-acetyl-glucosamine in mediating cell function and survival in the cardiovascular system. Cardiovasc Res.

[35] Gupta R, Jung E, Gooley AA, Williams KL, Brunak S, Hansen J (1999). Scanning the available *Dictyostelium*
*discoideum* proteome for *O*-linked GlcNAc glycosylation sites using neural networks. Glycobiology.

[36] Steentoft C, Vakhrushev SY, Joshi HJ, Kong Y, Vester-Christensen MB, Schjoldager KT (2013). Precision mapping of the human *O*-GalNAc glycoproteome through SimpleCell technology. EMBO J.

[37] Hamby SE, Hirst JD (2008). Prediction of glycosylation sites using random forests. BMC Bioinform.

[38] Li F, Li C, Wang M, Webb GI, Zhang Y, Whisstock JC (2015). GlycoMine: a machine learning-based approach for predicting N-, C- and O-linked glycosylation in the human proteome. Bioinformatics (Oxford, England).

[39] Zhang B, Li M-D, Yin R, Liu Y, Yang Y, Mitchell-Richards KA (2019). O-GlcNAc transferase suppresses necroptosis and liver fibrosis. JCI Insight..

[40] Bolanle IO, Riches-Suman K, Williamson R, Palmer TM (2021). Emerging roles of protein *O*-GlcNAcylation in cardiovascular diseases: insights and novel therapeutic targets. Pharmacol Res.

[41] Jensen ON (2004). Modification-specific proteomics characterization of posttranslational modifications by mass spectrometry. Curr Opin Chem Biol.

